# Identification of a novel gene in ROD9 island of *Salmonella* Enteritidis involved in the alteration of virulence-associated genes expression

**DOI:** 10.1080/21505594.2017.1392428

**Published:** 2018-02-27

**Authors:** Susmita Das, Shilpa Ray, Daniel Ryan, Bikash Sahu, Mrutyunjay Suar

**Affiliations:** School of Biotechnology, KIIT University, Bhubaneswar, Odisha

**Keywords:** *Salmonella* Enteritidis, SPI-1, ROD9, SPI-19, *SEN1005*, virulence, C57BL/6, inflammation

## Abstract

*Salmonella enterica* subsp. I serovar Enteritidis (*S*. Enteritidis), one of the causative agents for non-typhoidal gastrointestinal diseases in humans is an intracellular bacterium and mechanism for its invasion into host cells is critical to cause infection. The virulence of the pathogen is explained by the expression of genes located on its pathogenicity islands, mostly encoded under SPI-1 and SPI-2. However, *S*. Typhimurium SL1344, despite sharing ∼98% of its genome with *S.* Enteritidis P125109, lacks few regions of differences (ROD) that are hypothesized to impart virulence potential to *S.* Enteritidis. In this study, we created different mutants in the ROD9 island of *S.* Enteritidis, also referred as SPI-19 and identified a novel locus, *SEN1005*, encoding a hypothetical protein that is involved in its pathogenesis. Δ*SEN1005* displayed significantly reduced entry into cultured epithelial cells as well as uptake by macrophages and failed to cause acute colitis in C57BL/6 mice at day 3 post-infection (p.i.). Additionally, the global transcriptome analysis revealed a highly repressed SPI-1 and other down-regulated genes responsible for flagellar assembly, chemotaxis and motility in the mutant which correlated with decreased invasion and abated inflammation as compared to the wild-type. Therefore, our findings revealed that Δ*SEN1005* was attenuated *in vitro* as well as *in vivo* and we propose this hypothetical protein to play a role in altering the expression of genes involved in *Salmonella* virulence.

## Introduction

*Salmonella* infection is a global threat causing a wide range of clinical manifestations ranging from food poisoning to severe gastroenteritis (enterocolitis/diarrhea) and even systemic infections and enteric fever (typhoid). There are approximately 2500 serovars known for the two species and six subspecies of the bacteria and the degree of infection varies depending on the host resistance, type of serovars and alterations in expression levels of different virulent genes among these serovars.^1,2^ Serovars such as *S*. Typhi, *S*. Paratyphi and *S*. Sendai cause typhoid fever whereas many others including *S*. Dublin and *S*. Choleraesuis result in bacteremia in humans. However, serovars such as *S*. Typhimurium and *S*. Enteritidis have been reported to cause non-typhoidal food-borne gastroenteritis in humans as well as in mice model, cattle and chickens.^3–6^

*Salmonella* Enteritidis (*S*. Enteritidis) is a Gram-negative facultative intracellular pathogen and its virulence is mainly determined by two major pathogenicity islands, SPI-1 and SPI-2.^7^ These two islands encode for Type III secretion system (TTSS) 1 and 2 respectively and play an important role in delivering different effector proteins into the host that mediate pathogenesis.^8,9^ TTSS-1 and TTSS-2 have been extensively studied over the years and the effector molecules belonging to TTSS-1 promote invasion into epithelial cells and those included in TTSS-2 ensure bacterial replication in macrophages. During entry into host cells, these secretory proteins from the bacteria modify the actin cytoskeleton organization and temporary disarrangement of the membrane leading to access of bacteria into cells.^10^ Unlike other enteric pathogens, for example, *Vibrio cholerae* that causes secretory diarrhea, *Salmonella*, after crossing the intestinal epithelial barrier triggers various cytokines and chemokines causing inflammatory diarrhea which is characterized by the formation of submucosal edema, disappearance of goblet cells, loss of intestinal epithelial cell integrity, polymorphonuclear neutrophils (PMNs) infiltration in both humans and animals.^11^ Although the TTSS in *Salmonella* is considered to be a major component involved in the primary stage of causing infection, several other SPI and non-SPI genes have also been reported for contributing to virulence.^12–14^ Previous studies have shown the involvement of various transcriptional regulators from regions both inside and outside of these islands to control the expression of the genes associated to SPI-1 and SPI-2.^7^ Several genes such as InvF, HilA, HilC, HilD, RtsA positively regulate SPI-1 effectors whereas the nucleoid proteins H-NS and Hha behave as repressors.^15,16^ The ion channels, pili, fimbriae and flagella also play vital roles in interacting with the host cells and participate in the host-microbe cross-talk.^17,18^ *S.* Enteritidis can cause mucosal inflammation (colitis) either following the classical pathway, mediated by TTSS-1 or alternative pathway, which is dependent on TTSS-2. However, there are many other genes in the bacteria which might play a role in conferring virulence and hence need further characterization as the infection process in the host is never regulated by a single gene; rather an array of genes bearing various functions contributes to the pathogenesis. Although there is a ∼98.98% identity to the nucleotide sequence of the shared orthologs between *S*. Enteritidis strain P125109 and *S*. Typhimurium strain SL1344, the former has been shown to be more virulent over SL1344 in streptomycin pre-treated C57BL/6 mice in terms of higher bacterial burden in tissue and early inflammation kinetics.^19,20^ Earlier reports suggested that, on comparing the whole genome of both P125109 and SL1344, more than 200 genes were differentially distributed in either of the serovars, among which few genes belong to unique gene clusters present in *S.* Enteritidis, referred as regions of difference (ROD).^21^

In our investigation, a thorough analysis of *S.* Enteritidis genome reveals a few uncharacterized RODs which are assumed to be involved in higher virulence and survival of the bacteria. The ∼13.7 kb long island ROD9 of Enteritidis, also known as SPI-19 is a unique component comprising of hypothetical proteins that are absent in *S.* Typhimurium. Our *in silico* approach revealed few proteins in the ROD9 island of *S.* Enteritidis which have similar motifs with proteins involved in virulence of other bacteria such as *E. coli, Legionella, Vibrio, Pseudomonas*. Initial screening of the genes was performed by creating mutants in genes encoding those proteins of ROD9 through cell culture based infection assays that led to identification of a gene *SEN1005*, encoding a hypothetical protein to contribute to bacterial pathogenecity. Surprisingly, the isogenic mutant demonstrated attenuated phenotypes both *in vitro* and *in vivo*. Our study reports that this gene is immensely involved in invasion of *S*. Enteritidis into non-phagocytic cells as well as in its motility and is further found to be potentially associated in causing colitis in C57BL/6 mice at day 3 p.i. To understand the reason behind such attenuation, we compared the global transcriptomic expression between the wild-type (WT) and the mutant strain. About 227 genes were found to be differentially regulated in the mutant strain, of which the significantly down-regulated genes were primarily responsible for bacterial invasion and motility. All the observations led us to assume *SEN1005* to be a virulence determinant in *S.* Enteritidis and involved in the modulation of SPI-1 as well as other genes responsible for *Salmonella* pathogenesis.

## Results

### Δ*SEN1005* displays highly reduced invasion into epithelial cells

To exert its effect, *Salmonella* should first adhere and then invade the first line of host defense, the intestinal epithelium. For performing an initial screening, different genes were selected from the ROD9 island on the basis of protein blast (pBLAST) that showed similarity index to several virulent proteins belonging to other pathogens using Virulence Database (Table. S1). To determine the function of these genes in *S.* Enteritidis P125109, mutant strains viz. Δ*SEN0995*, Δ*SEN1002*, Δ*SEN1005*, Δ*SEN1008*, Δ*SEN1009* and ΔROD9 (Whole Island deleted) were created using the Lambda Red recombination system as described earlier.^22^

**Figure 1.**
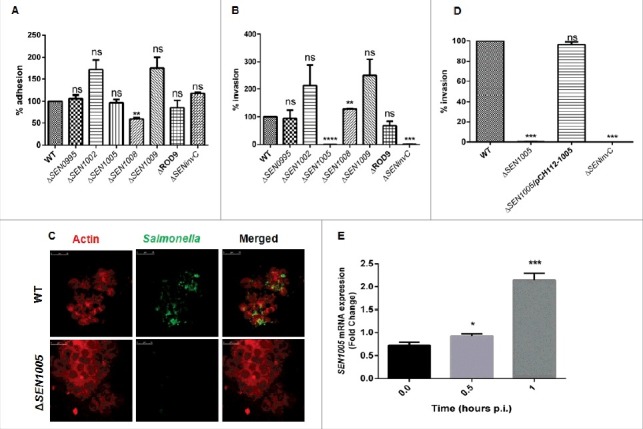
Bacterial Adhesion and Invasion assays in colon cancer epithelial cells HCT116. (A) Adhesion assay performed with WT and its ROD9 mutants in HCT116. (B) Invasion assay with WT and ROD9 mutants in HCT116. (C) Confocal microscopy to show invasion of GFP-expressing WT and Δ*SEN1005* in HCT116 with a MOI of 50 bacteria/cell. Cells were stained with Alexa Fluor 546 Phalloidin to detect actin in cytoskeleton. *Salmonella* shown in green and actin in red. Scale 10µm. (D) Invasion assay with WT, Δ*SEN1005* and Δ*SEN1005*/pCH112-1005 in HCT116 showing restoration of WT phenotype in the complemented strain. (E) Expression of *SEN1005* gene in HCT116 after infection with WT at indicated time points through qRT-PCR. Results were deduced from three independent experiments in triplicates and data represented as mean ± SD. ns, not significant (P>0.05); Statistical significance: *P < 0.05, **P < 0.01, ***P < 0.001 (Student's t-test).

There was no significant difference observed in adhesion of the mutants and the WT on the colon cancer epithelial lineage HCT116. However, Δ*SEN1008* exhibited 40% lesser adhesion as compared to WT (Fig. 1A).

To further demonstrate the ability of the mutants to cross the epithelial barrier in the host, invasion assay was performed in HCT116 cells. The results showed that, among the seven mutants used in the study, only Δ*SEN1005* was highly attenuated in invasion at 2 hours p.i. *in vitro* (Fig. 1B). Confocal imaging of HCT116 cells infected with Δ*SEN1005* and WT bacteria with GFP-expressing plasmid showed a significantly reduced invasion in cells infected with mutant as compared to WT infected cells (Fig. 1C). However, there was no significant alteration in the growth rate for both WT and the mutant strain (Fig. S1). The complemented strain Δ*SEN1005*/pCH112-1005 restored the WT invasion phenotype *in vitro* (Fig. 1D). Further, to understand the potential involvement of *SEN1005* in invasion, the expression of this gene was studied in the host cell after infection with WT *Salmonella* at indicated time points. It was observed that, there was a two-fold higher mRNA expression at 1 hour p.i. (Fig. 1E), indicating a possible role of the gene during invasion process. However, the expression levels decreased during later time points (data not shown).

### Δ*SEN1005* limits its uptake by macrophages

**Figure 2.**
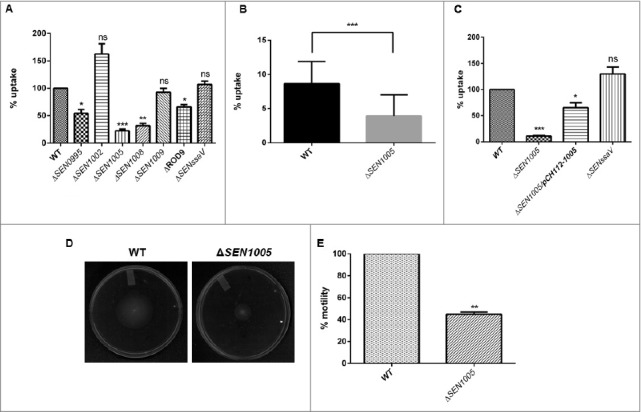
Bacterial uptake assay in murine macrophages RAW264.7 and analysis of motility. (A) % uptake of WT and its ROD9 mutants by murine macrophages RAW264.7 at 2h p.i.. (B) % uptake of GFP-expressing WT and Δ*SEN1005* by RAW264.7 at a MOI of 50 bacteria/cell after 2h of infection by flow cytometry. (C) Uptake of WT, Δ*SEN1005* and Δ*SEN1005*/pCH112-1005 by murine macrophages RAW264.7 at 2h p.i. that shows restoration of WT phenotype in the complemented strain. (D) Motility assay was performed with WT and Δ*SEN1005* by placing 1µl log phase cultures of bacteria with same O.D at the middle of 0.3% LB agar plates. After 5h of growth, the diameter of growth region was measured. Scale in cm. (E) Bar diagram for % motility of *Salmonella* measured from diameters of the bacterial growth. Results were deduced from three independent experiments in triplicates and data represented as mean ± SD. ns, not significant (P>0.05); Statistical significance: *P < 0.05, **P < 0.01, ***P < 0.001 (Student's t-test).

During systemic spread of *Salmonella*, macrophages play a vital role in transporting the bacteria to the tissues and allowing them to proliferate within the cells. Therefore, the potential to cause systemic infection can be evaluated from the ability of the macrophages to engulf bacteria. To examine this, all the seven mutants were used to determine their uptake by murine macrophage cells RAW264.7. Interestingly, there was 75% decrease in the uptake of Δ*SEN1005* as compared to the WT strain at 2 hours p.i. (Fig. 2A). The uptake assay was also performed with GFP-expressing WT and Δ*SEN1005* and results were analyzed by flow cytometry. A 50% lesser uptake of the mutant was noticed when compared to WT (Fig. 2B). The complemented strain restored the WT phenotype (Fig. 2C).

It has been reported that motility in bacteria is a key factor determining the uptake by macrophages as contact between the host cells and bacteria is necessary for the bacterial engulfment.^23,24^ Therefore, motility assay was performed for Δ*SEN1005* and WT on 0.3% LB agar plates and as expected, 50% diminished motility was observed in the mutant strain as compared to the WT after 5 hours of incubation (Fig. 2D and 2E).

Now, centrifugation of cells after infection with bacteria is a common method to enhance the contact between the host cells and the pathogen and this protocol has been used in several *in vitro* uptake experiments to resolve the differences in motility of variant strains.^24^ To delineate this phenomenon, uptake assay was performed with an additional experimental step where the cells were centrifuged at a lower speed (500xg) for 5 minutes just after infection, enabling a proper contact between the two, followed by usual incubation. Strikingly, the frequency of uptake was increased to 60% from 25% as observed in previous results. However, the uptake rate was still higher in the WT (Fig. S2 and 2A).

### Δ*SEN1005* fails to cause acute inflammation *in vivo* at day 3 p.i.

**Figure 3.**
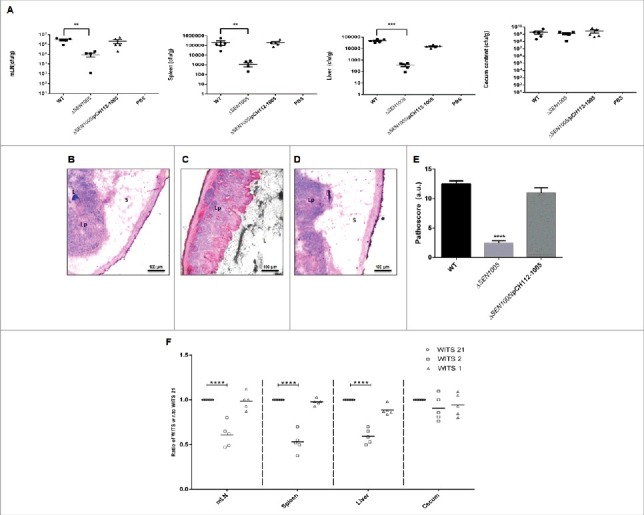
*In vivo* characterization of Δ*SEN1005*. Different groups of streptomycin-pretreated C57BL/6 mice were orally fed with ∼10^7^cfu of WT, Δ*SEN1005*, Δ*SEN1005*/pCH112-1005 and PBS (negative control) separately. (A) Bacterial dissemination: At day 3 p.i., mice were euthanized and the bacterial load was enumerated in mLN, spleen, liver and cecum by plating on MacConkey agar. (B-D) Tissue histopathology: Hematoxylin-Eosin stained images for sections of mice cecal tissue (5µm size) from WT, Δ*SEN1005* and Δ*SEN1005*/pCH112-1005 infected mice (left to right). Scale bar 100µm. S, submucosal edema; Lp, lamina propria; L, lumen. (E) Determination of cecal pathoscore based on the parameters described in materials and methods. (F) Assessment of competitive colonization by WITS- tagged WT (WITS21), Δ*SEN1005* (WITS2) and Δ*SEN1005*/pCH112-1005 (WITS1). Mice were infected with equal proportions of each strain with a total inoculum pool of ∼10^7^ cfu. At 72h p.i., the mice were sacrificed and their mLN, spleen, liver and cecum were collected and homogenized. The genomic DNA was isolated from each organ and the bacterial load of each strain was enumerated by qRT-PCR using WITS specific primers. The data was expressed as the ratio of WITS with respect to WITS21. Number of mice (n) = 5 for each group. Statistical significance: *P < 0.05, **P < 0.01, ***P < 0.001, ****P< 0.0001 (Student's t-tests for Fig. 3A and 3E; Two-way ANOVA for Fig. 3F).

Since, *S*. Enteritidis causes colitis in humans as well as in mice; we were interested to examine the degree of virulence of Δ*SEN1005* in streptomycin pre-treated C57BL/6 mice. After oral administration of *Salmonella* strains desired for the study, the colony forming unit (cfu) counts were assessed in fecal shedding of infected mice on day 1 and 2 p.i. A similar level of bacterial load was observed in all three groups of mice (WT, Δ*SEN1005 and* Δ*SEN1005*/pCH112-1005) indicating equal colonization in the gut (Fig. S3). On day 3 p.i., the bacterial loads (cfu) in the mesenteric lymph node (mLN) as well as in the systemic organs such as spleen and liver were found to be significantly higher in mice infected with WT and complemented strain, as compared to the mutant-infected group (Fig. 3A). To elucidate the cecal pathology, the Hematoxylin-Eosin (HE) stained cecal tissue sections of mice were carefully examined which showed almost negligible sign of inflammation in mutant-infected mice group by day 3 p.i., while WT and complemented strains caused severe colitis in mice showing loss of goblet cells, submucosal edema formation, epithelial cell disintegrity and PMN infiltration at the same time point (Fig. 3B–3D). As noted by the pathologists, the pathoscores of the cecal sections were 12.5 and 10.9 for the WT and complemented strain respectively, whereas Δ*SEN1005*-infected murine cecum was scored as 2.4 (Fig. 3E).

We further assessed the bacterial load in each tissue through a competition based mice infection assay where each mouse was orally administered with equal proportions of individually tagged WT, mutant and complemented strain (Fig. S4). The qRT-PCR results revealed significantly decreased colonization of systemic sites but almost similar cecal colonization by the mutant, in accordance with the previous *in vivo* findings (Fig. 3F).

### Δ*SEN1005* induces less production of pro-inflammatory cytokines and nitric oxide *in vitro*

**Figure 4.**
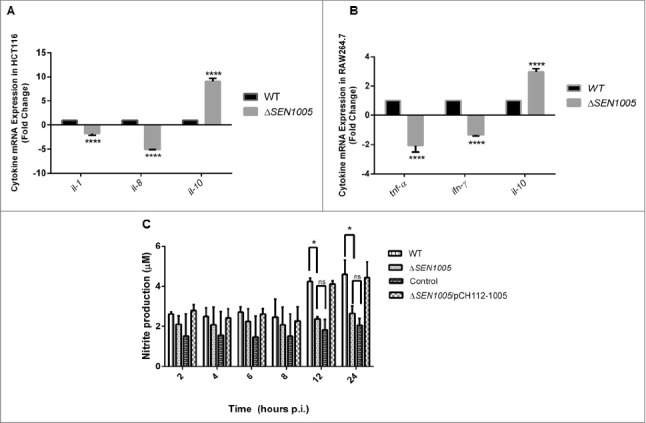
*In vitro* studies to show cytokine and nitric oxide production in epithelial and phagocytic cells after infection with WT and Δ*SEN1005.* (A) Expression profile of pro-inflammatory (IL-1 and IL-8) and anti-inflammatory (IL-10) cytokines in HCT116 by qRT-PCR. The data was presented corresponding to the fold-change differences in gene expression in Δ*SEN1005* with respect to WT. (B) RAW264.7 expressing TNF-α and IFN-ϒ (pro-inflammatory) and IL-10 through qRT-PCR. The data was presented corresponding to the fold-change differences in gene expression in Δ*SEN1005* with respect to WT*. gapdh* was considered as internal control. (C) Production of nitric oxide (nitrite) in RAW264.7 cells after infection with WT, Δ*SEN1005* and Δ*SEN1005*/pCH112-1005 at indicated time points by Griess reaction. Results were shown from three independent experiments in triplicates and data represented as mean ± SD. Statistical significance: *P < 0.05, **P < 0.01, ***P < 0.001, ****P < 0.0001 (Two-way ANOVA).

Cytokines are known to play crucial roles in regulating immune defense mechanism of the host by releasing pro- or anti-inflammatory signals after bacterial intervention.^25,26^ During *Salmonella* infection, host epithelial cells and macrophages produce various types of cytokines in response to bacterial attack.^27–29^ Further these cytokines (mostly pro-inflammatory), trigger the inflammation process in host which is mediated by the recruitment of macrophages and infiltration of polymorphonuclear neutrophils (PMNs) at the site of infection leading to damage in the cellular architecture of the host.^30^ We measured the mRNA levels of pro-inflammatory cytokines such as IL-1 and IL-8 in HCT116 as well as TNF-α and IFN-ϒ in RAW264.7 after infecting cells with wild type and mutant bacteria. The anti-inflammatory response in these two cell lines was examined by measuring the IL-10 levels. The qRT-PCR results showed a 1.6 fold and 9 fold reductions in the IL-1 and IL-8 cytokines in Δ*SEN1005* infected HCT116 cells (Fig. 4A). Similarly, the TNF-α and IFN-ϒ mRNA levels in the mutant infected RAW264.7 cells were found to be down regulated by 2 fold and 1.5 fold respectively (Fig. 4B). On the other hand, a significantly higher level of IL-10 was observed in both the cell lines infected with Δ*SEN1005* (9 fold in HCT116 and 2.45 fold in RAW264.7) (Fig. 4A and 4B). *gapdh* was taken as an internal control in all the experiments.

Enteroinvasive pathogens are reported to induce human cultured colon epithelial cells to produce nitric oxide (NO).^31^ NO is a marker of inflammation, a key component of innate immune system and is adequately produced by mammalian cells after infection with invasive strains of *E. coli, Salmonella, Shigella*. Also, a wide range of proteins have been reported to be up-regulated in RAW264.7 macrophages after *Salmonella* infection that includes proteins producing antibacterial NO.^32^ Additionally, NO production by macrophages has shown to be either cytostatic or cytotoxic for a wide range of invading pathogens, including *Salmonella*.^31^ Its production can be enhanced by the stimulation of cytokines such as IFN-ϒ, IL-1 or TNF-α in cultured cells.^33,34^ In our study, Greiss reaction was performed to estimate NO levels (Nitrite, the stable end product) in the culture supernatants after infecting RAW264.7 with either WT or mutant bacteria at different time points upto 24 hours p.i. (This time point was selected as the cells infected with WT survived for 24 hours). A gradual increase in the nitrite levels was noticed in the supernatants collected from WT infected macrophages with each passing hour and the level reached maximum at 24 hours. However, the mutant infected cells did not produce adequate amount of nitrites and their levels were almost similar at all time points of *Salmonella* infection. Particularly, at 12 and 24 hours p.i., the mutant infected macrophages produced significantly lower levels of nitrites as compared to cells infected with WT bacteria. The quantity of nitrites was minimal in the negative control while the complemented strain produced similar NO levels as WT (Fig 4C).

### Global transcriptome analysis of Δ*SEN1005*

**Figure 5.**
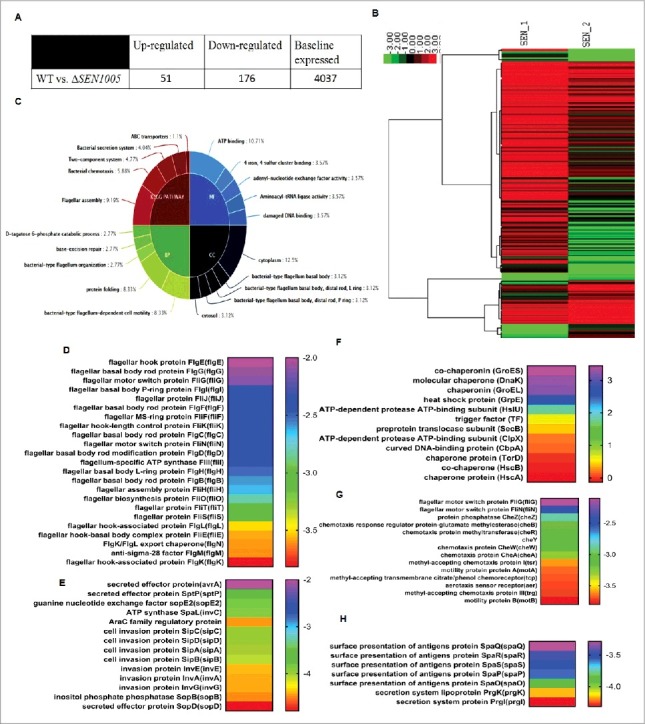
Global variations in gene expressions of WT vs Δ*SEN1005.* (A) Tabular representation of the number of genes either up-regulated or down-regulated in Δ*SEN1005* as compared to WT. (B) Hierarchical clustering of differentially expressed transcripts, SEN_1, WT; SEN_2, Δ*SEN1005.* (C) Top 5 GO and KEGG Pathway summary to illustrate various functional clusters that are differentially expressed in the two bacterial samples. (D-H) Heat maps showing differentially regulated genes in Δ*SEN1005* as compared to WT grouped according to their functions using David 6.7: (D) Flagellar assembly. (E) Epithelial cell invasion. (F) Protein folding. (G) Bacterial chemotaxis. (H) Bacterial secretion system. All the data sets were analyzed and differentially expressed transcripts were selected on the basis of RPKM >1 in either of the pair of samples with Log 2 fold change > = 2 with a t-test P Value <0.05.

Deletion of *SEN1005* encoding a hypothetical protein in SPI-19 of *S.* Enteritidis revealed drastic levels of attenuation both *in vitro* and *in vivo*. To further elucidate such phenotypes, we compared the global transcriptional profile of Δ*SEN1005* to the WT strain. Variations in expression between the two strains were determined by comparing the mean levels of expression of sample duplicates. Genes were accepted to be differentially expressed if the average fold change was either > = 2 or < = -2 with a t-test P Value <0.05 in order to maintain stringency on such huge data sets (see supplemental Table S3). As a result, 51/4264 (1.19%) genes were up-regulated while 176/4264 (4.12%) genes were down-regulated in Δ*SEN1005* relative to the WT (Fig. 5A). Global changes in gene expression are shown in the form of a heat map, SEN_1; WT and SEN_2; Δ*SEN1005* (Fig. 5B). To derive an in-depth knowledge of the differentially expressed genes, functional annotation and pathway analysis were performed with the help of GO,^35^ KEGG metabolic pathways^36^ (Fig. 5C) and DAVID 6.7^37,38^ (Fig. 5D–5H).

It is interesting to note that the expression of a wide range of genes contributing to virulence was found to be altered in the mutant. Several genes associated with bacterial chemotaxis and motility were repressed in Δ*SEN1005* (see Fig. 5G). Genes from SPI-1, directly related to invasion (see Fig. 5E) and other secretion system apparatus proteins required for surface presentation of antigens (Spa) (see Fig. 5H) were found to be highly down-regulated in the mutant. Another module found to be significantly affected in Δ*SEN1005* was that of flagellar assembly genes (Fig. 5D). These set of genes were remarkably repressed in addition to the genes encoding for motility. Apart from this, the chaperone family of proteins and a few proteases were found to be slightly up-regulated in the mutant (Fig. 5F).

However, many other clusters including the two-component systems, base-excision repair genes and those performing molecular functions were also found to be slightly modulated in the mutant (Fig. 5C and Table S3).

**Figure 6.**
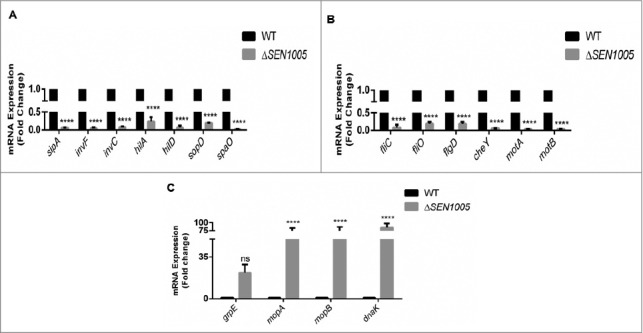
Validation of variable gene expression in WT and Δ*SEN1005* through qRT-PCR analysis. (A) Expression of SPI-1 genes in WT *vs* Δ*SEN1005* through qRT-PCR. (B) Expression of genes related to flagellar assembly, bacterial chemotaxis and motility in WT *vs* Δ*SEN1005* through qRT-PCR. (C) Expression of genes related to chaperone family of proteins in WT *vs* Δ*SEN1005* through qRT-PCR. The data was presented corresponding to the fold-change differences in gene expression in Δ*SEN1005* with respect to WT. Results were summarized from three independent experiments in triplicates and data represented as mean ± SD. ns, not significant (P>0.05). Statistical significance: *P < 0.05, **P < 0.01, ***P < 0.001, ****P < 0.0001 (Two-way ANOVA).

To validate our sequencing data, we performed qRT-PCR assays to determine the expression of few genes (from the transcriptome analysis) involved in the virulence process. The real-time analysis revealed an agreement in expression trends, albeit with different values due to the varying sensitivity between the two approaches (Fig. 6A 6C).

## Discussion

Non-typhoidal *Salmonella* infections pose serious global threats to human health and there is a need to identify and characterize bacterial proteins contributing to pathogenesis of the disease. Deletion of genes encoding such proteins in bacteria can be an approach to understand their roles in bacterial virulence and subsequently for making effective therapeutic intervention.^7^ Over decades, this issue has prompted researchers to carry out intensive study on the virulence potentials of different serovars and strains of this pathogen. Previous studies have shown bacterial motility and flagella to affect invasion by enhancing host-pathogen contact and further, the invasion-related bacterial TTSS-1, along with flagellar proteins is reported to elicit intestinal inflammation in various animal models.^24,39,40^ Apart from these, few RODs in *S*. Enteritidis P125109 strain have already been proved to be directly associated in causing salmonellosis in BALB/C mice.^14^ Although the major virulence determinants in *Salmonella* have been explored to examine their roles in promoting pathogenesis, many are yet to be characterized. Among the serovars which contain the differentially-distributed islands, the ROD9 (also called SPI-19) represents a truncated version in *S*. Enteritidis when compared with that in *S.* Gallinarum, *S.* Weltevreden, *S.* Dublin and *S.* Agona whereas this island is absent in *S.* Typhimurium.^41^ It is pertinent to note that there are two loci in ROD9 island of *S.* Enteritidis, termed as *SEN1001* and *SEN1002* which are already recognized as a part of an incomplete Type 6 secretion system (T6SS) and impart systemic colonization in BALB/C mice.^14^ We inferred that the remaining hypothetical proteins in this island may contribute to bacterial virulence.

To find a potential virulence factor, we first targeted the hypothetical proteins of SPI-19 that have some similarity index to virulent proteins of other Gram-negative bacteria. We could identify an unannotated gene *SEN1005*, downstream of the trimmed T6SS region, and found that the gene was involved in the invasion of the bacteria into non-phagocytic cells as well as in bacterial engulfment by the macrophages (Fig. 1D and Fig. 2C). It was also responsible for causing acute inflammation in C57BL/6 mice at 72 hours p.i. as observed by the pathoscores obtained from the HE-stained cecal sections (Fig. 3E). Using global transcriptomic analysis, we found Δ*SEN1005* strain to contain down-regulated invasion-associated genes and also those involved in bacterial flagellar assembly, motility and chemotaxis when compared to its WT. It is widely reported that all these genes in *S*. Enteritidis, *S.* Typhimurium and *S.* Gallinarum contribute to the initiation of infection and inflammation in mice or chickens.^40,42^ Motor switch proteins such as FliG and FliN, along with chemotaxis proteins namely CheY, CheZ help in signal transduction and determining the direction of flagellar rotation.^43^ Both chemotaxis and motility proteins such as MotA and MotB are shown to be involved in the invasion of *Salmonella* in the epithelial cell lineage, HeLa.^44^ Therefore, down-regulation of such a module would likely result in disrupted flagellar motility, non-invasiveness to both epithelial and macrophage cells and impaired protein transport activity. Further, SPI-1 genes of *Salmonella* have been reported to be critically involved in the invasion of epithelial cells and triggering an inflammatory response via the needle complex, TTSS-1.^2,17,45,46^ We observed a down-regulation of these genes in Δ*SEN1005* that possibly resulted in less invasion and poor inflammatory response *in vivo.* Significantly reduced levels of FliC have been shown to form an aflagellated *Salmonella* which further triggers low expression of IL-8, delaying early inflammation in the cecum.^39^ Thus, the repression of flagellar genes seen in the RNA-sequencing data as well as lower expression levels of both pro-inflammatory cytokines and nitrites in the mutant-infected cells *in vitro* (Fig. 4) might have contributed to a delayed immune response and failure to establish early colitis *in vivo*. Moreover, in the transcriptome data, the slight over expression of chaperonins suggested absence of any misfolded protein. Past studies have also shown DnaK and other few heat shock proteins such as MopA (GroEL) in *Salmonella* to act as adhesins and strengthen its ability to colonize mice.^47^ However, the ClpX protease has been earlier reported to repress the synthesis of flagellar regulon in *Salmonella*,^48^ indicating an impaired flagellum in the mutant, supporting our *in vivo* experiment that revealed Δ*SEN1005* capable of colonizing the gut of C57BL/6 mice efficiently, but unable to induce acute inflammation at 3 days p.i. The principal findings were hence correlated with the mRNA sequencing reports and qRT-PCR data showing SPI-1, flagellar, motility and chemotaxis genes to be highly attenuated and molecular chaperones to be up-regulated in the mutant strain.

The gene *SEN1005*,encoding a hypothetical protein consists of a M91 peptidase domain with a HEXXH motif, typical of a Zn metalloprotease.^49^ Previously, another effector protein, NleD identified in Enteropathogenic *Escherichia coli* (EPEC) strain had an identical peptidase domain showing catalytic activity to target the inflammatory response of the host.^50^ Additionally, other bacterial Zn metalloproteases have been of great importance in contributing to pathogenesis and invasion into hosts.^49,51^ Earlier reports have suggested that families of related metalloproteases from other bacterial species might be involved as toxic factors in causing infection as well.^52^ Although these proteases are usually considered to majorly perform proteolytic activities, it has been found that they can also act as regulators by modifying receptors and signaling molecules.^53,54^ In the current investigation, we tried to explore different virulent aspects of *SEN1005* in context of *Salmonella* infection. Based on our experimental observations and previous studies performed on Zn metalloproteases, we conclude SEN1005 to be immensely involved in virulence and that the absence of *SEN1005* led to drastic alterations in the expressions of SPI-1 and various virulence-related genes in the bacterium. Future studies are directed to evaluate the explicit mechanism on the roles of other proteins in the island with respect to pathogenesis and to dissect out the functional role of SEN1005 as a potential regulator, which can open up avenues to further understand the virulence determinants in *S.* Enteritidis.

## Materials and methods

### Bacterial Strains used in this study and Growth Parameters

The bacterial strains and plasmids used in the current study are listed in Table 1. All the strains were cultured in Luria-Bertani (LB) medium with antibiotics as required (streptomycin 50 µg/ml, kanamycin 50µg/ml, ampicillin 100µg/ml, chloramphenicol 20µg/ml). For SPI-1 inducing medium, LB broth was supplemented with 0.3M NaCl and the strains were cultured at 37°C at 120 rpm for 12 hours.^55^ For *in vitro* and *in vivo* infection experiments, overnight grown cultures were sub-cultured into fresh SPI-1 inducing medium to reach 0.6 O.D_600_.

**Table 1.** Bacterial strains and plasmids used in study.

Strains	Description	References
WT	*Salmonella enterica* serovar EnteritidisP125109	[3]
Δ*SEN0995*	*SEN0995*::Km (Mutant strain)	This study
Δ*SEN1002*	*SEN1002*::Km (Mutant strain)	
Δ*SEN1005*	*SEN1005*::Km (Mutant strain)	
Δ*SEN1008*	*SEN1008*::Km (Mutant strain)	
Δ*SEN1009*	*SEN1009*::Km (Mutant strain)	
ΔROD9	ROD9::Km (Mutant strain)	
Δ*SEN1005*/pCH112-1005	pCH112-1005 in *SEN1005*::Km (Complemented strain)	
*ΔinvC*	SEN*invC*::aphT (Mutant strain)	[3]
*ΔssaV*	*S.*EnteritidisΔ*ssaV*; Sm^r^	This study
Plasmids		
pKD4	Template plasmid; FRT-*aphT*-FRT (containing kanamycin resistance gene) bla FRT aph FRT PS1 PS2 oriR6K	[7]
pKD46	Red recombinase expression plasmid *bla*p*BAD gam bet exo*pSC101 *ori*TS (containing ampicillin resistance gene)	[7]
pCP20	FLP recombinase expression plasmid	[7]
pCH112	hilA ORF cloned into pBAD/myc-His; *ori*pBR322	[10]
pCH112-1005	*SEN1005* expressing vector*; SEN1005* cloned with its 1000bp upstream region by replacing HilA in *ori*pBR322 of pCH112	This study
pCJLA-GFP	GFP-plasmid used to tag strains	[58]

Bacterial cell propagation was examined for the three strains *viz.* WT, the mutant strain (Δ *SEN1005*) and the complemented strain (Δ*SEN1005*/pCH112-1005) to compare the growth rate of the same. For this purpose, bacterial cultures were collected at regular time intervals and serial dilutions were prepared to plate on LB agar for the enumeration of cfu.

#### Mutant preparation and development of plasmid-based complemented strain

In this study, we created deletion mutants using the Lambda Red recombination system where the genes were replaced by an antibiotic (kanamycin) resistance cassette using template plasmid pKD4 and pKD46 that contains the Red recombinase genes along with the arabinose-inducing promoter.^22^ All the primers used to create and confirm the mutations through PCR are listed in supplemental Table S2.

The complementation for *SEN1005* mutant was prepared by manipulating the plasmid pCH112.^10^ In brief, the *SEN1005* gene, along with its 1000bp upstream sequence was PCR amplified from *S*. Enteritidis P125109 genome and cloned in between NcoI and XbaI sites in the plasmid, replacing *hilA.* The clone was confirmed by PCR with insert specific primers and insert release, followed by transformation of the same in the Δ*SEN1005* strain.

#### Cell Adhesion and Invasion Assay

Cell adhesion and invasion assays were performed as reported previously.^56^ Briefly, the HCT116 cell line was cultured in Dulbecco's modified Eagle's medium (DMEM) (Gibco), supplemented with 10% Fetal Bovine Serum (FBS). Cells were seeded at the density of 1 × 10^5^ per well and incubated for 24 hours prior to infection to reach 80% confluency. From overnight grown bacterial cultures, subculture (1:20 dilution) was done under SPI-1 inducing conditions (0.3 M NaCl LB, 37°C, 120 rpm) and the O.D_600_ was adjusted to 0.6. Bacterial cultures were then diluted in LB at 1:5 ratio. HCT116 cells were washed with 1X PBS and DMEM (without antibiotics) was added. The cells were then infected with 5µl of the diluted culture to obtain a MOI (multiplicity of infection) of 10 bacteria/cell, centrifuged at 500xg for 5 minutes ensuring proper host-microbe contact and were further incubated for another 50 minutes in 5% CO_2_ incubator for invasion assays. Next, the media was exchanged with DMEM (Gibco), containing 100µg/ml gentamicin to kill the extracellular bacteria. At 2 hours p.i., cells were lysed with 0.1% sodium deoxycholate diluted in 1X PBS to release the intracellular bacteria and serial dilutions of the lysed cells were plated to obtain the number of bacteria that has invaded the cells. For adhesion assay, both the inoculums and 24 well plates were kept on ice for 15 minutes prior to infection. Infection was then done with bacterial cultures at a MOI of 10 bacteria/cell and was incubated for another 30 minutes on ice, immediately followed by lysis, serial dilution and plating. Each assay was performed thrice in triplicates.

#### Macrophage Uptake

Macrophage uptake assay was done as described earlier.^57^ In short, the murine macrophage cell line RAW264.7 was cultured in DMEM (Gibco), supplemented with 10% FBS. Cells were seeded at the density of 1 × 10^5^ per well and kept for 24 hours prior to infection. Overnight grown bacterial cultures were washed with 1X PBS and diluted to 1 × 10^8^cells/ml in DMEM (without antibiotics). RAW264.7 cells were then infected with 20µl of diluted bacterial culture to obtain a MOI of 10 bacteria/cell and then incubated for the next 50 minutes in 5% CO_2_ incubator. Next, the media was replaced with DMEM (Gibco), containing 100µg/ml gentamicin to kill the extracellular bacteria. At 2 hours p.i., cells were lysed with 0.1% Triton X-100 (HIMedia) and serial dilutions of the lysed cells were plated to obtain the number of bacteria phagocytosed by the macrophages. The experiment was performed thrice in triplicates.

#### Confocal microscopy

Confocal microscopy was performed to show invasion of *Salmonella*, following a protocol described earlier.^58^ The HCT116 cells were seeded in DMEM (Gibco), supplemented with 10% FBS at 50,000 cells/cover slip inside each well of a 24 well plate and incubated overnight in a humidified chamber at 37°C overnight with 5% CO_2_. Next day, invasion assay was performed as mentioned earlier but with WT and mutant *S*. Enteritidis containing plasmid pCJLA-GFP that constitutively expresses GFP and using a MOI of 50 bacteria/cell. After infection, the cover slips were washed with 1X PBS thrice, fixed with 4% paraformaldehyde (PFA) (Sigma-Aldreich), blocked with 5% BSA, stained with Alexa Flour 546 Phalloidin (Invitrogen) and then mounted on slides with Antifade reagent (Invitrogen Molecular Probes).The slides were visualized using Confocal Laser Scanning Microscope, Leica TCS SP5 under 63X oil immersion objective. The invading bacteria were observed by Z-series scanning.

#### Animal Ethical statement

The animal model experiment was permitted by the Institutional Animal Ethics Committee (IAEC) and all the experiments were performed abiding the strict rules and regulations set by the board facilitating minimum suffering of the animals involved. The approval number for the same is KSBT/IAEC/2014/MEET-1/A11.

#### Mouse infection and Histopathological Study

The animals were housed in the animal house facility of School of Biotechnology, Bhubaneswar, Odisha, India for 7 days to acclimatize with the environment. The strains WT, Δ*SEN1005*, Δ*SEN1005*/pCH112-1005 were grown in SPI-1 inducing media for 12 hours, followed by subculture (1:20) for 4 hours to obtain ∼10^7^cfu. For this study, streptomycin-pretreated mouse model was used as described.^7^ Four groups, each having 5 mice were fed with either of the mentioned strains and PBS (as negative control) using oral gavages. Feces were collected at 1 and 2 days p.i. to check for colonization of the bacteria. At 3 days p.i., all the mice were sacrificed to enumerate the amount of bacteria present in the cecum, mesenteric lymph node (mLN), spleen and liver after homogenizing the tissues and plating on Mac Conkey agar plates, supplemented with appropriate antibiotics. Cecal tissues were cryopreserved and 5 µm sections were prepared, HE-stained and evaluated for pathological score by two pathologists on considering the histopathological parameters like formation of submucosal edema, disappearance of goblet cells, infiltration of polymorphonuclear neutrophils (PMNs) and epithelial cell disintegrity. The pathoscores were determined based on the mentioned parameters out of a total 13 arbitrary units (a.u.) corresponding to different degrees of inflammation that include healthy intestines showing nil inflammation (0); negligible or very minute inflammation (1–2); slight inflammation (3–4); medium inflammation (5–8); and acute inflammation (9–13).

#### Preparation of tagged strains of WT and *Δ1005 S*. Enteritidis, competitive mouse infection assay and qPCR

To generate WITS, *S.* Enteritidis WT was tagged with specific barcode sequences as described previously.^59^ The mutant strain was made kanamycin sensitive with the help of the pCP20 flip-recombinase plasmid to obtain a clean mutant (only streptomycin resistant) prior to tagging. WITS-21 was used to tag the WT strain whereas WITS-1 and WITS-2 were tagged separately to the mutant strain by conventional phage transduction procedure described earlier.^3,7^ Δ*SEN1005* tagged WITS-1 strain was further transformed with pCH112-1005 to generate the complemented tagged strain. The colonies bearing WITS tags were selected on LB agar plates supplemented with 50µg/ml kanamycin and confirmed with PCR using primers specific to individual unique tags and a common primer to *ydgA*, located near the integration site. An additional PCR with *SEN1005* specific primers was done to confirm the absence of the gene in the mutant strain after tagging. Competitive mouse infection study was performed following the procedure mentioned earlier.^60^ Briefly, the three WITS tagged strains were grown individually in LB overnight, followed by subculture under SPI-1 inducing conditions up to O.D_600_ 0.6 and infected to 5 mice in 1:1:1 ratio, the total inoculum pool equaling to ∼10^7^ cfu. 3 days p.i., the mice were sacrificed to collect mLN, spleen, liver and cecal content and genomic DNA was isolated from each organ using DNeasy Blood & Tissue Kits (Qiagen) and qRT-PCR was done using WITS specific primers. The relative abundance of different bacterial strains was calculated in terms of ratio of WITS-1 and WITS-2 with respect to WITS-21 (i.e. WT). All the primers used in this experiment are listed in Table S2.

#### Motility assay

Bacterial swimming motility was measured on 0.3% w/v soft LB agar plates. Strains were grown overnight, adjusted to same O.D and inoculated at the centre of the plate as a drop. After 5 hours of incubation at 37°C, the spread of the bacterial colony was assessed by measuring the diameter of the bacterial halo.^55^ The experiment was repeated thrice.

#### Flow Cytometry

Macrophage uptake assay was performed as mentioned in RAW264.7 with the WT and mutant strain (Δ*SEN1005*), after transformation of each with GFP-expressing plasmid pCJLA-GFP. The cells that engulfed the bacteria expressed GFP whose intensity was quantified by FACScanto™ II flow cytometer.

#### NO estimation

WT and Δ*SEN1005* bacterial cultures grown overnight were used to infect murine macrophage cell lines RAW264.7 at a MOI of 20 bacteria/cell. Centrifugation at 500 xg for 5 minutes was done post-infection to ensure host-pathogen contact. The infected cell lines were incubated for an hour to facilitate bacterial entry. Upon incubation, the monolayers were washed thrice with 1X PBS and incubated for next 24 hours in fresh DMEM supplemented with 50µg gentamicin to kill and stop invasion of any residual extracellular bacteria. After desired time intervals, culture supernatants were collected, centrifuged at 12,000xg for 15 minutes at 4°C to remove any cell debris and stored for further use. NO is highly unstable and is usually converted to stable end products like nitrite and nitrate. The nitrite present in the supernatant was estimated by performing Griess reaction.^61^ In brief, 100µl of the supernatant was treated with Griess reagent (0.1% sulphanilamide followed by 0.01% napthylethylene diamine, both dissolved in 2.5% H_3_PO_4_) (Sigma-Aldrich). The mix was incubated at room temperature for 15 minutes in dark conditions. Finally, the absorbance was taken at 550 nm and data was analyzed by plotting a standard curve using different known concentrations of sodium nitrite.

#### RNA extraction and Real-Time PCR Assays

From overnight grown cultures of *S*. Enteritidis WT and Δ*SEN1005* strains, subculture was done in SPI-1 inducing media to reach O.D_600_ at ∼0.6. For RNA sequencing, the subcultured bacteria were snap-frozen in liquid nitrogen before RNA isolation. RNA was extracted using Trizol reagent (Ambion), followed by RNase free DNaseI treatment (Thermo Fisher Scientific) and cDNA synthesis using kit from HIMedia. With the diluted and normalized cDNA templates, quantitative RT PCR was performed using Kapa Sybr Fast qPCR Master Mix (2x; Kapa Biosystems, USA). Either 16s rRNA or Guanylate monophosphate kinase (*gmK*) genes was used as housekeeping genes for the bacterial samples and *gapdh* for mammalian samples in the qRT-PCR assays. The qRT-PCR data were presented corresponding to the fold-change differences in gene expression in Δ*SEN1005* with respect to WT. Experiments were repeated thrice in triplicates. All the primers used are listed in supplementary Table S2.

For infection experiments, a MOI of 50 bacteria/cell was used and after 2 hours p.i. the mammalian cells were harvested and immediately processed for RNA extraction, subsequent purification, cDNA synthesis and qRT-PCR analysis as already discussed.

### cDNA library construction, sequencing and analysis

cDNA libraries of the WT and ΔSEN1005 strains were prepared in duplicate and sequenced (paired end) on an Illumina Hiseq sequencer at Bionivid Technology Pvt. Ltd. (India). Raw data quality control was performed using the NGS QC Toolkit to ensure that at least 70% of the total bases per read had a Phred quality score of 20 or higher. Bacterial transcriptome analysis was performed using Rockhopper62 with reads mapped to the genome of S. Enteritidis P125109 (Acc No. AM933172.1) with the average number of HQ reads profiled to WT and ΔSEN1005 being 32.58 and 31.04 million respectively and average read length of 101, followed by annotation using the prokaryotic genome annotation tool (PGAT).63 All the data sets were analyzed and differentially expressed transcripts were selected on the basis of RPKM >1 in either of the pair of samples with Log 2 fold change > = 2 with a t-test P Value <0.05. Further downstream analysis was performed using Top 5 GO, David 6.7 and KEGG metabolic pathways to cluster genes based on function and identify co-regulated genes.

#### Statistical Analysis

All the experiments were done at least thrice in triplicates and graphs were plotted showing mean and standard deviation. The data were statistically verified with Student's t-tests and ANOVA using GraphPad Prism version 6.0.

## Supplementary Material

New_folder__2_.zip
