# Supplementary material for: Identification of a novel gene in ROD9 island of Salmonella Enteritidis involved in the alteration of virulence-associated genes expression
Source: Virulence. 2018 Feb 27;9(1):348–62. doi: 10.1080/21505594.2017.1392428 (PMC5955183; doi:10.1080/21505594.2017.1392428)
Supplement: New_folder__2_.zip [file kvir-09-01-1392428-s001.zip › New folder (2)/2017VIRULENCE0174R1-s02.docx]

| **Gene locus** | **Protein Annotated in NCBI**  **Supplementary Tables** | **Protein Domain/Motif** | **Similarity to virulent proteins in other bacteria using Virulence Database** |
| --- | --- | --- | --- |
| SEN0995 | Hypothetical protein | YCII-related domain | - |
| SEN0996 | Hypothetical protein | - | - |
| SEN0998 | Hypothetical protein | DUF1311 | - |
| SEN0999 | Hypothetical protein | PHA02239 | - |
| SEN1000 | Hypothetical protein | - | - |
| SEN1001 | DNA-binding protein | LysM domain | - |
| SEN1002 | Hypothetical protein | DUF796 | Hcp1 family type VI secretion protein in *Pseudomonas*, *Vibrio* |
| SEN1003 | Hypothetical protein | ImpA_N domain | Type VI secretion protein in *E.coli* |
| SEN1005 | Hypothetical protein | Peptidase_M91 | *E. coli* effector, NleD |
| SEN1006 | Hypothetical protein | - | - |
| SEN1007 | Hypothetical protein | - | - |
| SEN1008 | Hypothetical protein | Sel1 repeat | Related to TPR repeats, *Legionella* enhanced entry protein EnhC |
| SEN1009 | Hypothetical protein | Sel1 repeat | - |
| SEN1010 | Transposase | Transposase;Homeodomain-like domain;Helix-turn-helix domain | - |
| SEN1013 | Lipoprotein | dsRNA gated channel septation initiation-1 | - |
| SEN1013A | Hypothetical protein | - | - |
| SEN1013B | Hypothetical protein | - | - |

**Table S1.** Tabular representation of gene loci that belong to *Salmonella* Enteritidis ROD9 island ranging from *SEN0995* to *SEN1013B*. pBLAST of ROD9 proteins was performed against Virulence database showing similarity to other virulent bacteria.

| **Primer** | **Sequence (5’-3’)** | **Description** | **Reference** |
| --- | --- | --- | --- |
| FwSipA | TCCAGTTGCGGGCCTTTAAT | SPI-1 effectors | [55] |
| RwSipA | GCAGACCGAGATCAAAACGC |  |  |
| FwSopD | TGGTTCGAAGATGACCTGGC |  |  |
| RwSopD | AGTGAGTCCTGCCATTCGAC |  |  |
| FwInvF | TCCACTAATCCTGCGCCATC | Regulators controlling SPI-1 expression |  |
| RwInvF | CCGTTGTCGCACCAGTATCA |  |  |
| FwHilA | GGGCAGATGATACCCGATGG |  |  |
| RwHilA | AAGAGAGAAGCGGGTTGGTG |  |  |
| FwHilD | TGACAAATACCCGGCAGCAA |  |  |
| RwHilD | AGTTTACCGCTCCGAAAGCA |  |  |
| FwInvC | AGATACAGGTGTCCGTCAAG | ATP synthase | This study |
| RwInvC | GTTATTGACCTGTGGCGTAG |  |  |
| FwSpaO | ACCAGTTCGCCATTACCCAG | Surface presentation of antigens protein | This study |
| RwSpaO | ACAGAGCGACCGTTTGAGTT |  |  |
| FwFliC | TCGCGTAGTCGGAATCTTCG | Flagellar assembly genes | [57] |
| RwFliC | CTTTGGCACAGGTTGACACG |  |  |
| FwFliO | TCGACCTCGACAATCACCAC |  | This study  This study |
| RwFliO | AAAGGAAATAGCGTCCGGGG |  |  |
| FwFlgD | TTGGCGTGGAATTGCAACAG |  |  |
| RwFlgD | TACCGTTGTTCCGTCCGTTT |  |  |
| FwMotA | GTATTCTGGCCGATGCGGTA | Bacterial motility and chemotaxis genes |  |
| RwMotA | CTGCTGCTGGTTTGGGTTTC |  |  |
| FwMotB | AGCAGCCGAATATCGACGAG |  |  |
| RwMotB | CGATCGGCGGATAATTCCCA |  |  |
| FwCheY | ATCTCCGACTGGAACATGCC |  |  |
| RwCheY | CTGCGGTGAACGGTTTTACG |  |  |
| FwGrpE | CAATCACTTCCACGCCGAAC | Bacterial chaperone and heat shock proteins | This study |
| RwGrpE | GTCGCAGACAAAGCCAATCC |  |  |
| FwMopA | ACTACGGTTACAACGCAGCA |  |  |
| RwMopA | ATCAGACCAGCCACAGAAGC |  |  |
| FwMop**B** | ATCATCGCTGTCGGTAAGGG |  |  |
| RwMopB | ATGTCGCCAACTTTCACGTC |  |  |
| FwDnaK | TCTTTGGTAAAGAGCCGCGT |  |  |
| RwDnaK | AGACTGGTTGTCTTCCGCAG |  |  |
| Fw 16srRNA | TTCCAGTGTGGCTGGTCATC | Housekeeping genes for *Salmonella* | This study |
| Rw 16srRNA | TGCCTGATGGAGGGGGATAA |  |  |
| Fwgmk | AGCAAATTCGCGAAAAGATG |  | [55] |
| Rwgmk | TGGCAATGACTTCTTCGCTAT |  |  |
| Fw 0995 KO | CGTTGTTCTGGTGTGAACTCCGACTGTCGGGCTTTGTTGTTTTTGGTGTGTAGGCTGGAGCTGCTT | SEN995gene mutation; Km cassette amplification | This study |
| Rw 0995 KO | CATAGAGTGCTAGCATAATATCCCCTTTATATTTATGGACAACAAAATATGAATATCCTCCTTAGT |  |  |
| Fw 1002 KO | TTGTAAAATTTTTACTAAAACACCCTCTCATCCCATAAACTGAATTGTGTAGGCTGGAGCTGCTT | SEN1002gene mutation; Km cassette amplification |  |
| Rw 1002 KO | CCGAAAATAATAGTTAAATCAAGGAGATTAACATGGCCAATTTAATATATGAATATCCTCCTTAGT |  |  |
| Fw 1005 KO | ATGTCTGCCAGAAGGCATTCAGTTCTGTACGCCAGTCGTCATTTTTGTGTAGGCTGGAGCTGCTT | SEN1005gene mutation; Km cassette amplification |  |
| Rw 1005 KO | TCAGTATTTCATCCTTGGTGGGATTCCCATTTCTTTGCGGACAGGGTTATATGAATATCCTCCTTAGT |  |  |
| Fw 1008 KO | AGTAAGAAATCCATCTATTTTAACAATAGTAAGCTGAAGGATAACTGTGTAGGCTGGAGCTGCTT | SEN1008gene mutation; Km cassette amplification |  |
| Rw 1008 KO | GTCCCAGATTATAAATAGCAACTGGATTATTGGATTCAGCAGCCTATATGAATATCCTCCTTAGT |  |  |
| Fw 1009 KO | AATCCAGTTGCTATTTATAATCTGGGACACATCTATAATTATGGATGTGTAGGCTGGAGCTGCTT | SEN1009gene mutation; Km cassette amplification |  |
| Rw 1009 KO | AGTAGAATGATCTTCTGTCGCTGAAAAATCAAGCATCTTCAATAAATATGAATATCCTCCTTAGT |  |  |
| Fw ROD9 KO | CGTTGTTCTGGTGTGAACTCCGACTGTCGGGCTTTGTTGTTTTTGGTGTGTAGGCTGGAGCTGCTT | ROD9island deletion; Cm cassette amplification |  |
| Rw ROD9 KO | CTTAGTCTGAGAGGAATAAAACCGGCTGATGAATGACTCAAGATGATATGAATATCCTCCTTAGT |  |  |
| Conf. 995 | AAATTGAAAGCCGGATGGTGGTGTTATT | Confirmatory primers for knock outs |  |
| Conf. 1002 | GAGGGTGTCCAGACCCTTTC |  |  |
| Conf. 1005 | TATCGCCCACGAAATGGGGC |  |  |
| Conf. 1008 | CGGAGTGGCAACTGTTGCAG |  |  |
| Conf. 1009 | AGGCTGCTGAATCCAATAATCCA |  |  |
| Conf. ROD9 | AAATTGAAAGCCGGATGGTGGTGTTATT |  |  |
| Km/Kt | CGGTCCGCCACACCCAGCC |  | [55] |
| WITS-1 | ACGACACCACTCCACACCTA | Confirmatory primers for WITS tagging | [3] |
| WITS-2 | ACCCGCAATACCAACAACTC |  |  |
| WITS-21 | ACAACCACCGATCACTCTCC |  |  |
| ydgA-Fw | GGCTGTCCGCAATGGGTC |  |  |
| FwpCH 1005NcoI | AAACAACCATGGACGACCGGATTGTCATACGG | Cloning primers for  SEN1005complementation | This study |
| RwpCH 1005XbaI | AATGCTTCTAGATCAGTATTTCATCCTTGGTGGGATTC |  |  |
| Fw 1005 | AGGTGACTTTTAAGGGTGACGAAGAACAGC | Real-time primers for SEN1005expression |  |
| Rw 1005 | TATATTCGGAGGGATCGTAGCATGTATGTT |  |  |
| Fw IL-1β | AGGCTCATCTGGGATCCTCT | Pro-inflammatory human cytokines |  |
| Rw IL-1β | CATCACTGTCAAAAGGTGGCA |  |  |
| Fw IL-8 | TCTGTCTGGACCCCAAGGAA |  |  |
| Rw IL-8 | CCAAAAAGGCAGATACCTAATGACG |  |  |
| Fw TNF-α | TTCTCAAAATTCGAGTGACAAGCCT | Pro-inflammatory mouse cytokines |  |
| Rw TNF-α | GTACAACCCATCGGCTGGC |  |  |
| Fw IFN-ϒ | ATTCAGAGCTGCAGTGACCC |  |  |
| Rw IFN-ϒ | GGAAGCACCAGGTGTCAAGT |  |  |
| Fw humanIL-10 | CCGTGGAGCAGGTGAAGAAT | Anti-inflammatory human cytokines |  |
| Rw humanIL-10 | GCCTATTGAGTCCCACCACC |  |  |
| Fw mouseIL-10 | TGGGTGAGAAGCTGAAGACC | Anti-inflammatory mouse cytokines |  |
| Rw mouseIL-10 | GTCCAGCAGACTCAATACACAC |  |  |
| Fw hGAPDH | AGGGCCCTGACAACTCTTTT | Housekeeping genes for human and mouse |  |
| Rw hGAPDH | AGGGGTCTACATGGCAACTG |  |  |
| Fw mGAPDH | GAGAGGCCCTATCCCAACTC |  |  |

**Table S2:** Primers used in study

**Supplementary Figures**

**Figure S1.** Growth curve experiment to check the growth pattern in WT, Δ*SEN1005* and Δ*SEN1005*/pCH112-1005 in the form of cfu counting at different time intervals till 9 hours. The experiment was performed thrice in triplicates and data represented as mean ± SD.

**Figure S2.** Uptake assay of WT, Δ*SEN1005* by murine macrophages RAW264.7 with an added centrifugation step at 500xg for 5 minutes immediately after infection to ensure proper contact between bacteria and host cells. The experiment was performed thrice in triplicates and data represented as mean ± SD (Student’s t-test).

**Figure S3.** Fecal shedding of different *Salmonella* strains from streptomycin-pretreated C57BL/6 mice (n=5) orally fed with ~10^7^ cfu of WT, Δ*SEN1005*, Δ*SEN1005*/pCH112-1005 and PBS (negative control) separately as analyzed by plating at day 1 and day 2 p.i.

**Figure S4.** Assessment of pre-inoculum density by qPCR. *S*. Enteritidis was tagged with WITS as follows: WT (WITS 21), Δ*SEN1005* (WITS 2) and Δ*SEN1005*/pCH112-1005 (WITS 1). Genomic DNA was isolated from the mix inoculum pool and proportion of each strain was obtained by qPCR with WITS specific primers. The data was represented as ratio of WITS 2 and WITS 1 with respect to WITS 21 (WT) present in the inoculum.
